# Tomato detection based on modified YOLOv3 framework

**DOI:** 10.1038/s41598-021-81216-5

**Published:** 2021-01-14

**Authors:** Mubashiru Olarewaju Lawal

**Affiliations:** Institute of Agricultural Engineering, Shanxi Agricultural University, Jinzhong City, 030801 Shanxi China

**Keywords:** Computer science, Computational science

## Abstract

Fruit detection forms a vital part of the robotic harvesting platform. However, uneven environment conditions, such as branch and leaf occlusion, illumination variation, clusters of tomatoes, shading, and so on, have made fruit detection very challenging. In order to solve these problems, a modified YOLOv3 model called YOLO-Tomato models were adopted to detect tomatoes in complex environmental conditions. With the application of label what you see approach, densely architecture incorporation, spatial pyramid pooling and Mish function activation to the modified YOLOv3 model, the YOLO-Tomato models: YOLO-Tomato-A at AP 98.3% with detection time 48 ms, YOLO-Tomato-B at AP 99.3% with detection time 44 ms, and YOLO-Tomato-C at AP 99.5% with detection time 52 ms, performed better than other state-of-the-art methods.

## Introduction

The application of artificial intelligence to agriculture has pulled in increasingly more consideration around the world particularly in harvesting robots’ development. This harvesting robot was introduced to replace manual picking of fruits that is very tedious, time-consuming, expensive, and relatively high in human error. Meanwhile, the autonomous detection of fruits or other agricultural products is the first important step for harvesting robots. Based on the detection accuracy results, a manipulator is usually guided to pick the fruits. However, the development of a computer vision system that is intelligent as humans for fruit and vegetable detection is very difficult. This is because of numerous reasons, for example, occlusion, uneven illumination, nonstructural fields, and other unpredictable factors^1^.

Over the years, serious endeavors have been made in fruit detection for the harvesting robots. Yin et al.^2^ employed the L * a * b* color space to extract ripe tomatoes, and Wei et al.^3^ used segmentation based on color to extract fruits from its background. However, it is very hard to choose the best color model in real life situation^3^, because the color features extraction for fruit detection intensely depend on the effectiveness of used color space. The analysis of shape technique for mature apples’ localization reported by Kelman et al.^4^ noted an influenced of illumination and leaves on the performance. Zhao et al.^5^ recorded 93% accuracy on segmented mature tomatoes from background using an optimal threshold on fusion image features. Due to the adoption of only features, their obtained results were affected by the illumination.

The quest for artificial intelligence improvement led to machine learning research for computer vision tasks in agriculture. Lu et al.^6^ detailed an accuracy of 92.4% for branch and fruit identification in natural scenes based on only RGB trained with support vector machine (SVM). The obtained results outperformed threshold-based methods. Nevertheless, the outcomes were prone to be influenced by illumination. For mature tomato detection, Liu et al.^7^ applied false color removal technique and SVM on coarse-to-fine framework. Despite the fact that the reported Recall and Precision individually accomplished 90.00% and 94.41%, the technique is not satisfactory for occluded and overlapped tomatoes. According to Liu et al.^8^, majority of the methods in machine learning depends on handcrafted features. Handcrafted features are complex to design, can only adjust to some particular conditions, and have low-level abstraction. This resulted into a weak flexibility and its possess transfer of methods difficulty from one kind of fruit to several others. The drawbacks of traditional machine learning were conquered after the introduction of deep learning on computer vision^9^.

The computer based statistical model created by deep learning with convolutional neural networks can attain state-of-the-art accuracy, sometimes exceeding human-level performance with proven outstanding in image classification^10^, segmentation, and object detection^11^. The model is trained with a large set of labeled data and neural network architectures that contain many layers^10^. A successful high precision has been reported on technologies to agriculture compared to the traditional machine-learning approaches^9,12^. Sa et al.^13^ combined multi-modal color (RGB) and Near-Infrared (NIR) information to experiment on Faster R-CNN^14^ detector for fruit detection. Although relatively small number of images were used for training and testing, this method obtained better results (F_1_ score = 80.7% to 83.8%) than previous methods. However, it is difficult for the method to detect small fruits, and its speed still requires improvement for real-time in-field operation of harvesting robot. The modified Inception-ResNet architecture^15^ applied by Rahnemoonfar et al.^16^ for fruit counting achieved an average accuracy of 91% with real images. Nevertheless, the method did not implement detection, only counted fruit. The fruit detection model in orchards proposed by Bargoti et al.^17^ based on the Faster R-CNN reported more than 90% of F_1_ score as most of the missing fruits came from the case where fruits appear in tight clusters.

**Figure 1:**
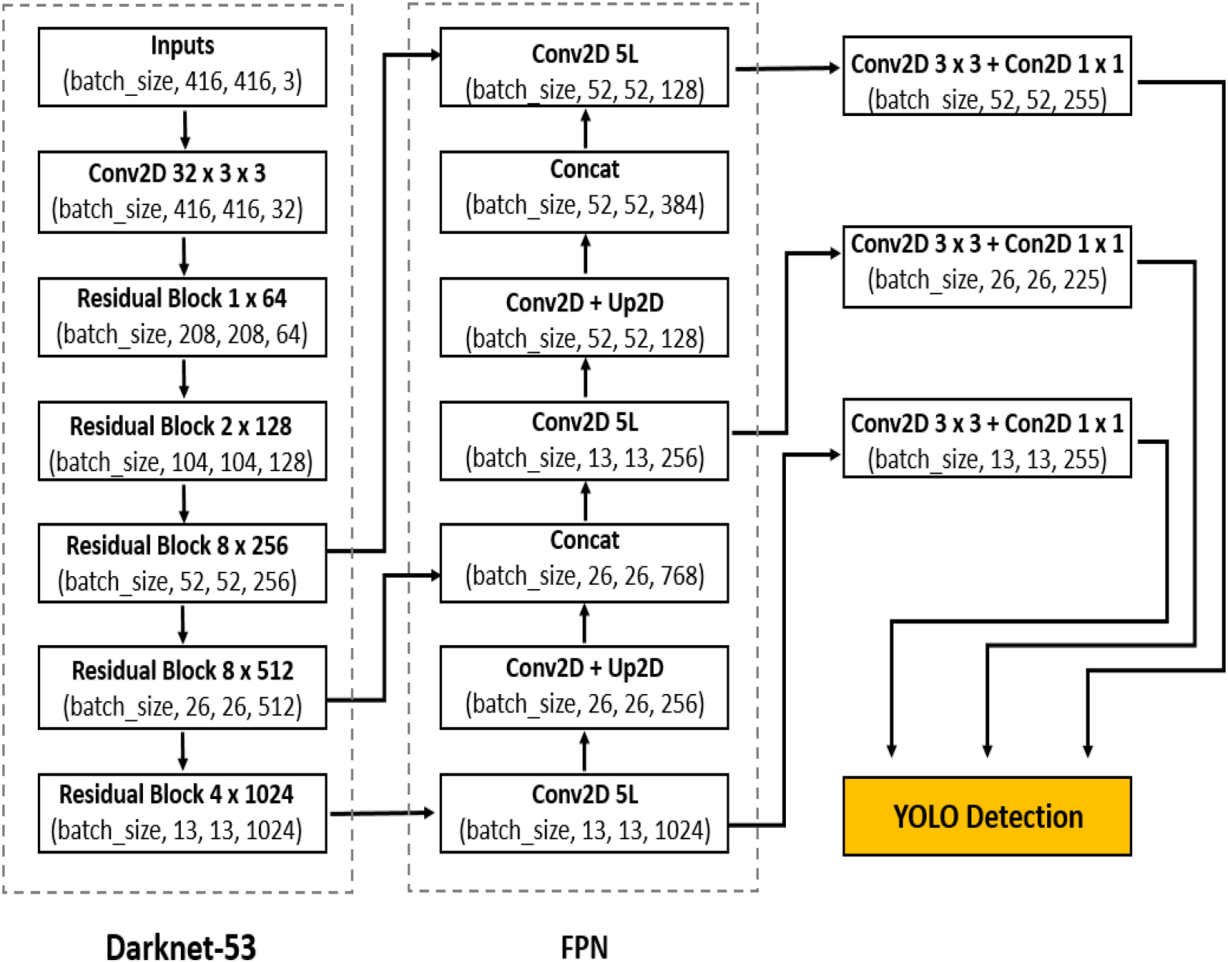
YOLOv3 architecture.

You Only Look Once (YOLO) models was proposed by Redmon et al.^18–20^ for object detection. Its combines the region proposal network (RPN) branch and classification stage into a single network, leading to more concise architecture, state of the art performance in object detection with high computation speed and better computational efficiency, making them the true sense of real-time detectors. YOLO models directly predict the bounding boxes and their corresponding classes with a single feed forward network compared with previous region proposal based detectors^14,21^ that perform detection in a two-stage pipeline. YOLOv2^19^ is the second version of YOLO^18^ that was proposed with the objective of improving the accuracy significantly, while making it faster. The idea of anchors for detection introduced into YOLOv2 was inspired by Faster R-CNN. The anchors improve detection accuracy, simplify problem and ease the learning process of the network. Meanwhile, batch normalization^22^ was added to the convolution layers to push mAP to 2% and also skip connection^23^. YOLOv2 significantly improves localization and Recall compared to YOLO. YOLOv3^20^ became one of the state-of-the-art for object detection as a build on YOLO and YOLOv2. YOLOv3 uses multi-label classification, binary cross-entropy loss for each label instead of using mean square error in calculating the classification loss. YOLOv3 predicts objects in three different scales (similar to feature pyramid network(FPN)^24^) as shown in Fig. 1 and the score for each bounding box using logistic regression. DarkNet-53 (YOLOv3 backbone) is used to replace the DarkNet-19 as a new feature extractor. The whole DarkNet-53 network is a chain of multiple blocks with some strides 2 convolution layers in between to reduce dimension. Each of the block contains bottleneck structure of 1 × 1, followed by 3 × 3 filters with skip connections similar to ResNet. DarkNet-53 possesses less billion floating point operations (BFLOP) compared to ResNet-152, but achieves 2 × faster with the same classification accuracy. YOLOv3 shows significant improvement for small objects detection and performs very well with speed involvement. YOLOv4 next version to YOLOv3 was introduced recently by Alexey et al.^25^. Its runs twice faster than EfficientDet with comparable performance. YOLOv3’s AP and FPS was improved by 10% and 12%, respectively in YOLOv4. YOLOv4′s framework is composed of CSPDarkNet53 as a backbone, spatial pyramid pooling (SPP)^26^ additional block, path aggregation network (PANet) as neck^27^ and YOLOv3 head. CSPDarkNet53 enhance the learning capacity of CNN with Mish^28^. The SPP is added over the CSPDarkNet53 to significantly increase the receptive field, separates out the most important context features and causes almost no reduction of the network operation speed. PANet is used for the collect of feature maps from different stages in YOLOv4 instead of the FPN used in YOLOv3. YOLOv4 enables widespread adoption of conventional GPU with an improve accuracy of the classifier and detector.

Real‑time mango detection in orchard reported by Koirala et al.^29^ obtained F_1_ score of 96.8%. Furthermore, Liu et al.^8^ proposed a new circular bounding box (C-Bbox) for tomato detection by replacing the rectangular bounding box (R-Bbox) which was tested on YOLOv3 framework. An improved result of 93.91% and 96.4% were respectively reported for AP and F_1_ score for YOLO-tomato. It was also proven in the report that illumination and occlusion factors are solvable with YOLOv3 algorithm. However, there are few literatures on tomato detection based on modified YOLOv3 with densely architecture and SPP incorporation, and most published papers uses large dataset that are later preprocessed. This requires great amount of time, labor costs and better hardware in image data collection, labeling, and training. For a computer vision system to be as intelligent as humans, then it must be treated as human.

This study adopts a modified YOLOv3 model called YOLO-Tomato models to detect tomatoes in complex environment conditions by using label what you see (LWYS) technique. The ideas proposed to limit the drawbacks in deep learning and to make detector as intelligent as humans, include the use of small dataset obtained from complex environment condition, label what you see approach, the incorporation of densely architecture^30^ into YOLOv3 to facilitate reuse of features for well generalize tomato detection and SPP application to reduce missed detections and inaccuracies. The main purpose is to increase the variability of the input images, so that the designed tomato detection model has higher robustness to the images obtained from different environments. The experiments demonstrated that the proposed method can achieve a high detection accuracy including real-time detection speed under uneven environment.

## Methods

### Dataset construction

**Figure 2:**
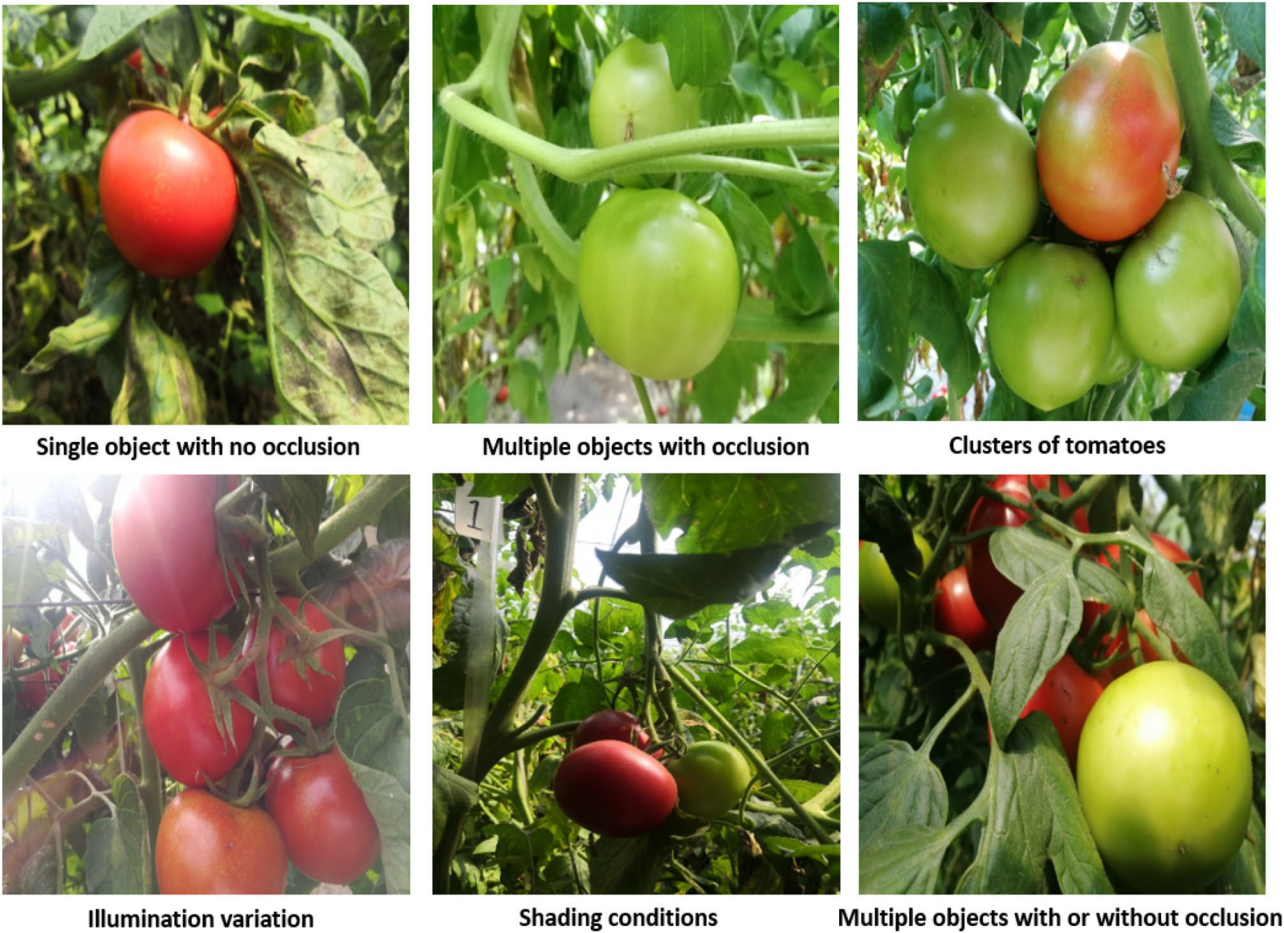
Tomato samples under different growing environments: (**a**) single object with no occlusion, (b) multiple objects with occlusion, (**c**) clusters of tomatoes, (**d**) illumination variation, (e) shading conditions, and (**f**) multiple objects with or without occlusion.

The tomato datasets used in this research work were collected from Taigu, Jinzhong, China. The best operational distance between the camera and tomato trees in field, that is 0.5–1.0 m for harvesting robot was used. The images were taken using a digital commercial camera, with a 3968 × 2976-pixel resolution, RGB color space and JPG storage format. All the images were captured under natural daylight conditions, including complexity of the growing environments: illumination variation, occlusion, and overlap^8^. This increases significantly the difficulty of tomato detection (ripe and unripe) in the field. For deep learning simplicity, a total of 125 tomato images were captured and divided into 80% of training set and 20% test set. Randomly, each of the captured images comprised of single object with no occlusion, single object occluded by branches and leaves, multiple objects with or without occlusion and so on. Some image samples from the created dataset under different environments are shown in Fig. 2.

To investigate tomato detection performance via resizing influence, all images were resized to 0.5 and 0.25 according to the aspect ratio of the original(Raw) images. This is to maintain the original image aspect ratio. The datasets of tomato were grouped into Raw, 0.5 ratio and 0.25 ratio for training and testing.

Labelled data are required for YOLO detection models training, i.e. the class-label and position (co-ordinates) of all ground truth bounding boxes in training images^18–20^. While labelling is manual and labor intensive process, annotation i.e. the drawing of ground truth bounding boxes was easier, because the number of created dataset in each category are small. This reduces chances of human error. The graphical image annotation tool labelImg (https ://github.com/tzutalin/labelImg) was used to hand label all the ground truth bounding boxes, with annotation files saved in YOLO format^20^.

**Figure 3:**
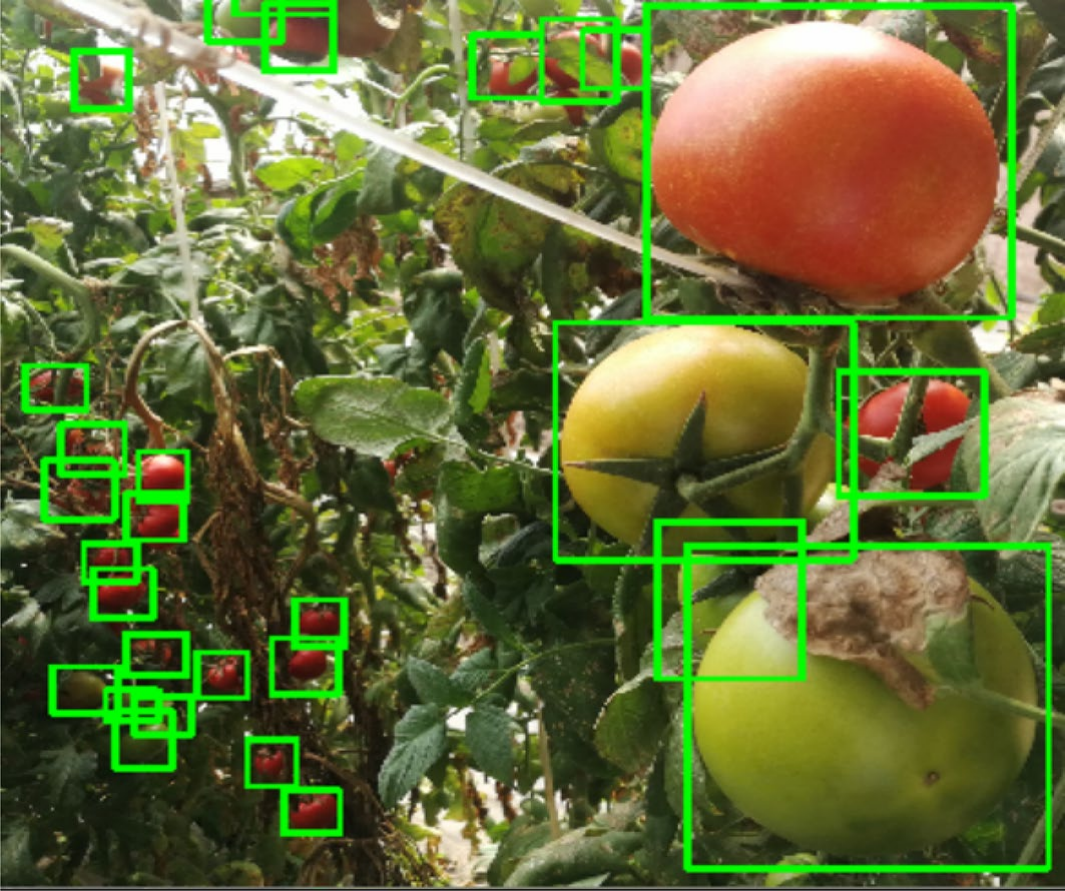
Label what you see (LWYS) technique on Tomato image.

In each image, all the visible tomatoes for ripe and unripe were labelled with a bounding box based on LWYS technique. Notably, for the highly occluded tomatoes, the bounding boxes were drawn by the supposed shape depending on the visible part of humans’ intelligence (Fig. 3). After that, the annotated images were checked three times by different people to ensure that no unannotated class was missing out.

### YOLO-tomato model

**Figure 4:**
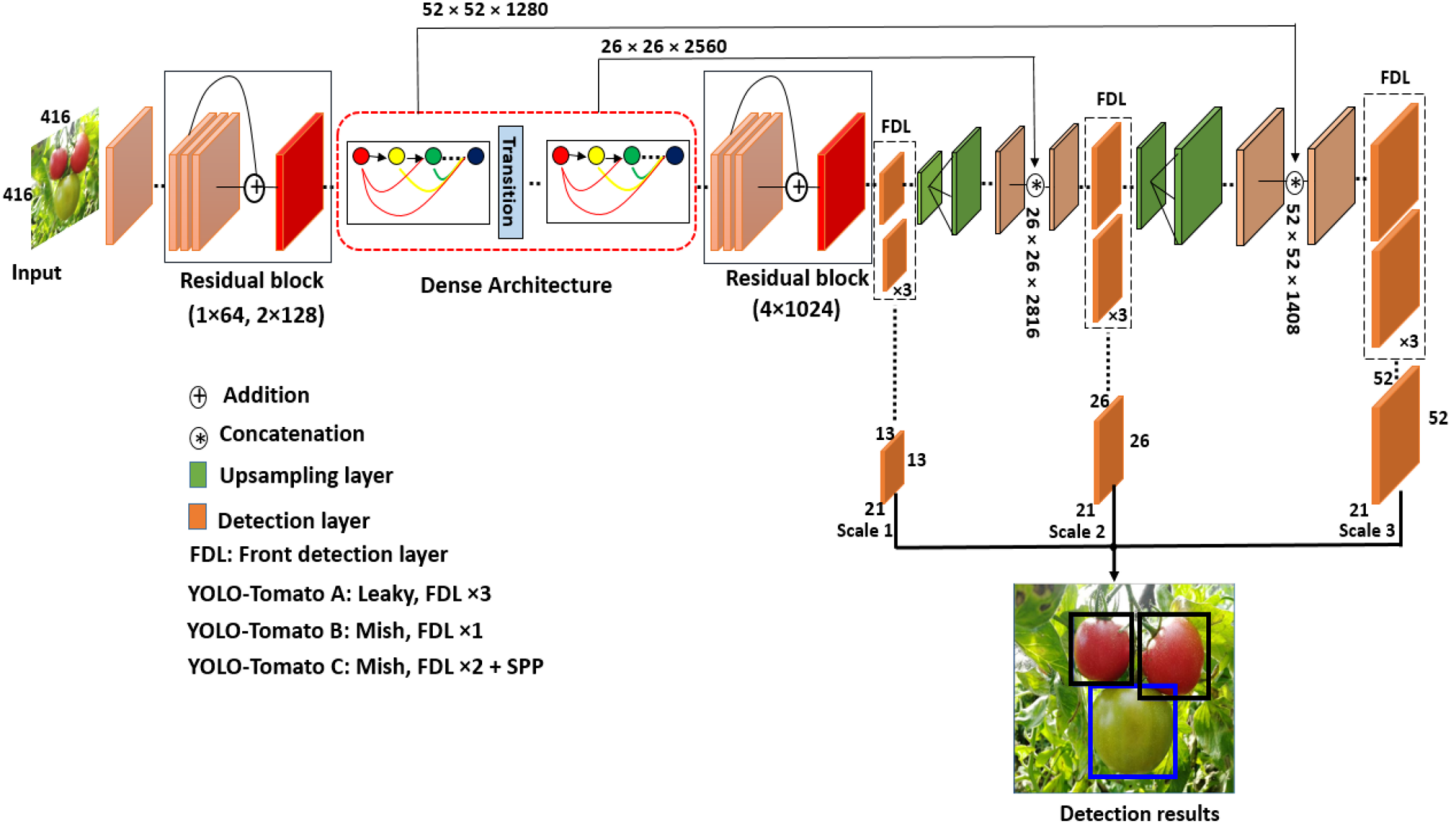
Overview of YOLO-Tomato model.

**Figure 5:**
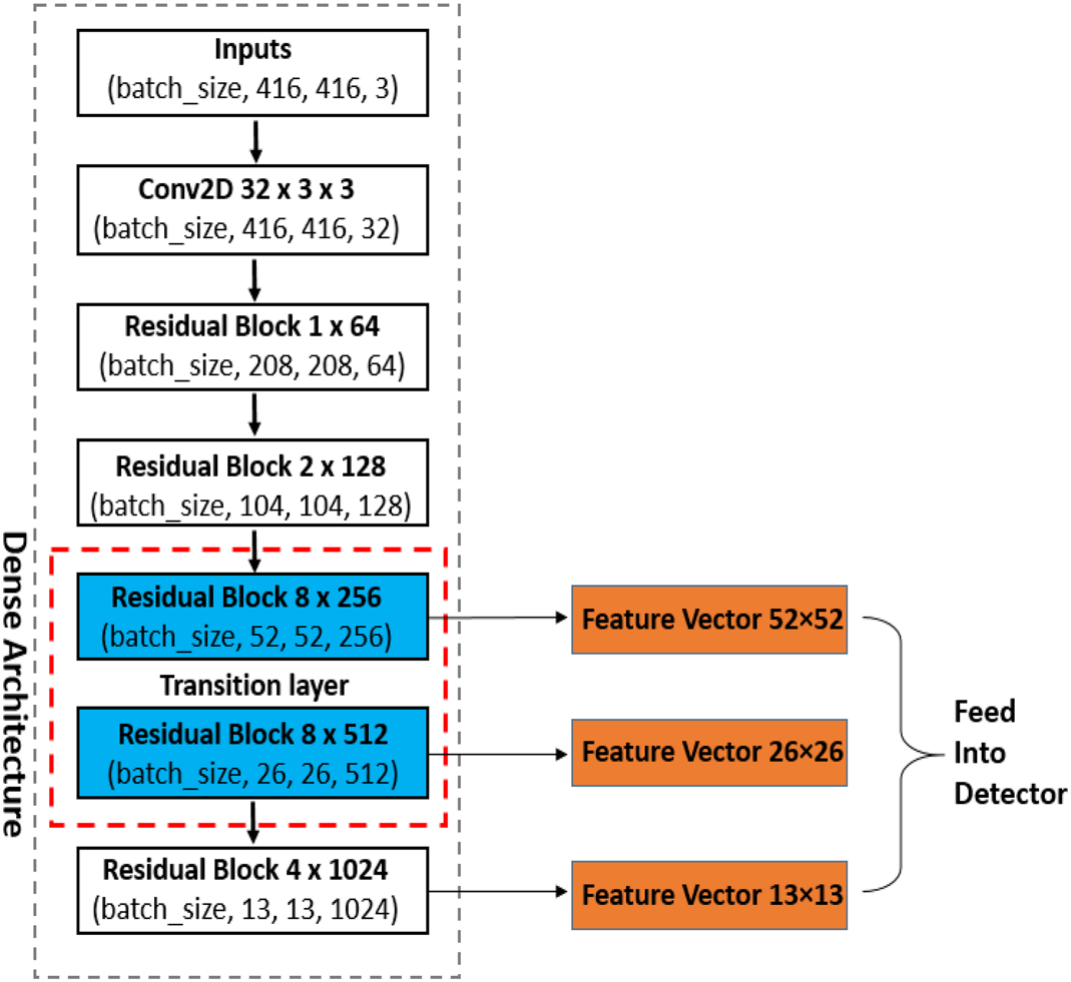
DenseNet architecture position in YOLO-Tomato model.

Based on the YOLOv3 architecture shown in Fig. 1, a densely connected architecture proposed by Huang et al.^30^ was incorporated for better feature reuse and representation. This enables more compact and accurate models for detection^30^. An overview of the modified tomato detection model is shown in Fig. 4 for 2 classes (Ripe and Unripe tomato). The design of YOLO-tomato model replaced the residual block 8 × 256 and residual block 8 × 512 in YOLOv3 (Fig. 1) with dense architecture arrangement^30^ shown in Figs. 4 and 5 (blue color). This is to enhance a deeper network within the detection scale outlet. A 1 × 1 bottleneck layer^23^ and 3 × 3 convolutional layer were stacked together for each dense layer^30^. A transition layer was placed between the two dense layers in order to make the model more compact^30^. The main rationale behind the modifications was to enable detection on multiple feature maps from different layers of the network. This would allow accurate detection of smaller tomato under different environment. With all things being same as YOLOv3 model in Fig. 1 including its loss function^20^, the concatenated features of 26 × 26 × 768 increases to 26 × 26 × 2816 and 13 × 13 × 384 increases to 13 × 13 × 1408 features in the FPN of YOLO-tomato model. The increased features of YOLO-tomato help to preserve more fine grained in detecting smaller tomatoes to fit into LWYS method.

Furthermore, the YOLO-tomato model was divided into YOLO-tomato-A, YOLO-Tomato-B, and YOLO-Tomato-C. This is to study the effects of different activation functions and front detection layer (FDL) reduction towards building a YOLO-tomato real-time detection model that is accurate and faster. YOLO-Tomato-A was activated with Leaky Rectified Linear Unit (ReLU)^31^ having FDL × 3. The six layers of YOLOv3 were pruned as YOLO-Tomato-B was activated with Mish^28^ having FDL × 1, and YOLO-Tomato-C was activated with Mish^28^ having FDL × 2 and SPP^26^. Mish defined as: *f*(*x*) = *x*⋅*tan*ℎ(ς(*x*)), where ς(*x*) = ln(1 + *ex*) is the softplus activation function^28^ was reported to have outperform ReLU defined as: *f*(*x*) = max (0, *x*)^31^. This activation function plays an important role in the performance of every deep neural network by introducing non-linearity^28^.

**Figure 6:**
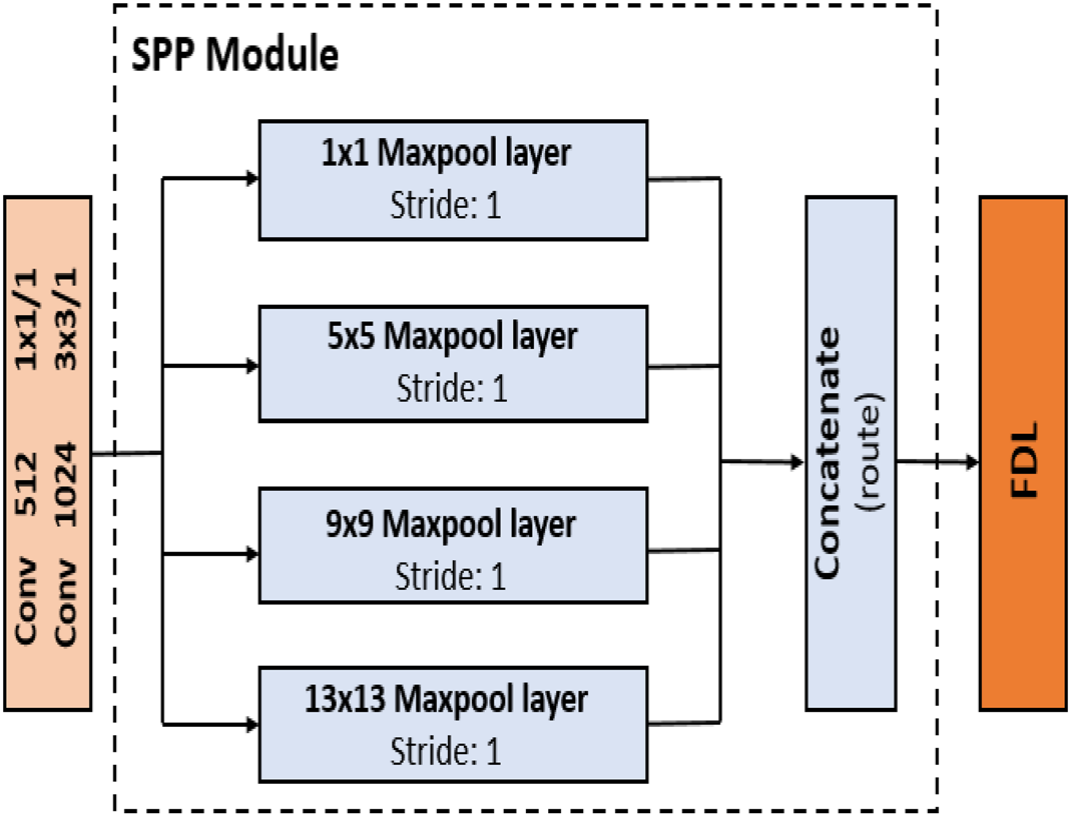
Spatial pyramid pooling module.

The idea of SPP^26^ was introduced after the last residual block (i.e. residual block 4 × 1024) of the YOLO-Tomato-C to optimize the network structure. As the convolutional layers deepened, the receptive field of a single neuron is gradually increasing, the extracted feature capability is enhanced with more abstract during the feature extraction process^32^ of the YOLO-Tomato-C. Nevertheless, the position information of the small target becomes inaccurate or even lost in severe cases^32^ if the shape of the tomato’s feature map is blurred. With the large number of tomatoes in the images, missed detections and reduced accuracies will happen. Therefore, SPP module in Fig. 6 can solve the problem. According to Huang et al.^32^, it is a feature enhancement module, which extracts the main information of the feature map and performs stitching.

### Experimental platform and evaluation

**Figure 7:**
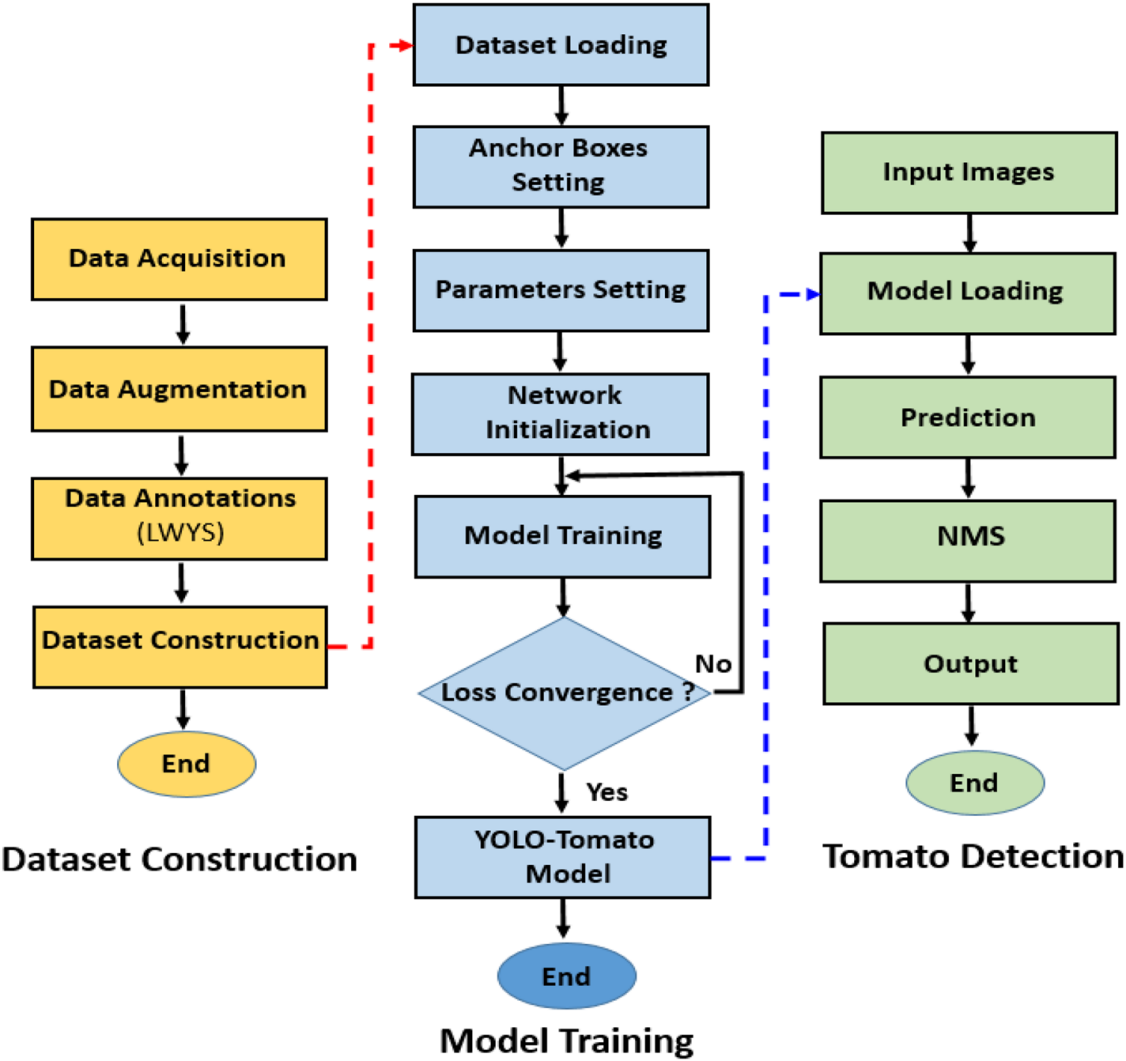
YOLO-Tomato model flowchart for dataset, training and detection process.

Model training and testing was implemented on a computer with the following specifications: i7-8700 CPU 64-bit 3.20 GHz, 16 GB RAM, NVIDIA Quadro M4000 GPU, CUDA v10.2, cuDNN v7.6.5, OpenCV v4.2.0. Figure 7 provides a detail flowchart of dataset, training and detection process of YOLO-Tomato model used in this study.

Before training and testing, it is important to find the size of the anchor box that is most likely to be counted from the constructed dataset, instead of using the default anchor box configuration provided by YOLOv3 to create too specialized predictors. The K-mean clustering algorithm was used to generate 9 clusters at 416 × 416 pixels according to 3 scales of detection layer shown in Fig. 5. The anchors were arranged and assigned in descending order to each scale to improve the YOLO-tomato models. Because the datasets of tomato were categorized into Raw, 0.5 ratio and 0.25 ratio, three different 9 clusters were generated. The obtained results of average IoU show that Raw is 77.45%, 0.5 ratio is 78.33% and 0.25 ratio is 78.55%.

The model receives inputs images of 416 × 416 pixels. The adjustment of the learning rate reduces training loss^20^. The learning rate was chosen to be 0.001 between 0 and 4000 iterations with maximum batches of 4000, because the input images contains two classes (ripe and unripe tomato). In order to reduce the memory usage, the Batch and Subdivision were respectively set to 64 and 16. The momentum and weight decay were set to 0.9 and 0.0005, respectively. Furthermore, random initialization approach was used to initialize the weights for training the YOLO-Tomato, while the official pre-trained weights was used for YOLOv3 and YOLOv4.

To verify the effectiveness of the conducted experiments on the trained YOLO-tomato, YOLOv3, and YOLOv4 models, Precision, Recall, F_1_-score and AP are used as evaluation parameters. The calculation method is shown in Eqs. (1)–(4).

1 $$ {\text{Precision = }}\frac{{{\text{TP}}}}{{\text{TP + FP}}} $$

2 $$ {\text{Recall = }}\frac{{{\text{TP}}}}{{\text{TP + FN}}} $$

3 $$ {\text{F}}_{{1}} { = }\frac{{{2 } * {\text{ Precision }} * {\text{ Recall}}}}{{\text{Precision + Recall}}} $$

In these equations, TP, FN, and FP are abbreviations for True Positive (correct detections), False Negative (missed detections), and False Positive (incorrect detections). F_1_ score was conducted as a trade-off between Recall and Precision to show the comprehensive performance of the trained models^8^, defined in Eq. (3). Average Precision–AP^33^ was adopted to show the overall performance of the models under different confidence thresholds, expressed as follows:

4 $$  {\text{AP}} = \sum\limits_{{\text{n}}} {\left( {{\text{r}}_{{{\text{n}} + 1}}  - {\text{r}}_{{\text{n}}} } \right)\mathop {\max }\limits_{{\tilde{{\text{r}}}:\tilde{{\text{r}}}^{3} {\text{r}}_{{{\text{n}} + 1}} }} {\text{p}}\left( {\tilde{{\text{r}}}} \right)}   $$

where ${\text{p}}\left( {{\tilde{\text{r}}}} \right)$ is the measured Precision at Recall ${\tilde{\text{r}}}$.

## Results and discussion

### Model performance

The trained models were tested using the image resolution of 416 × 416 pixels set at batch size 1 in order to maintain consistency with the training image resolution. The YOLO-tomato models detect number of tomatoes in the test dataset achieving good detection results. The Precision, Recall, F_1_-score and AP of the detected tomatoes were calculated and compared with YOLOv3 and YOLOv4 model. The experimental results are shown in Tables 1 for Raw, Table 2 for 0.5 ratio and Table 3 for 0.25 ratio.

**Table 1:** Model performance evaluation with Raw dataset under 416 × 416 pixels’ resolution.

Methods	Activation	FDL	Precision (%)	Recall (%)	F_1_ (%)	AP (%)
YOLOv3	Leaky	× 3	97.4	96.2	96.8	97.8
YOLOv4	Mish + SPP	× 3	97.4	100.0	98.7	99.5
YOLO-Tomato-A	Leaky	× 3	96.1	99.3	97.7	98.2
YOLO-Tomato-B	Mish	× 1	96.2	99.4	97.8	99.3
YOLO-Tomato-C	Mish + SPP	× 2	97.0	99.3	98.1	99.5

**Table 2:** Model performance evaluation with 0.5 dataset under 416 × 416 pixels’ resolution.

Methods	Activation	FDL	Precision (%)	Recall (%)	F_1_ (%)	AP (%)
YOLOv3	Leaky	× 3	94.4	98.0	96.2	97.3
YOLOv4	Mish + SPP	× 3	98.0	99.9	99.0	99.5
YOLO-Tomato-A	Leaky	× 3	95.7	99.5	97.6	98.5
YOLO-Tomato-B	Mish	× 1	94.5	99.1	96.7	99.3
YOLO-Tomato-C	Mish + SPP	× 2	95.8	99.9	97.8	99.5

**Table 3:** Model performance evaluation with 0.25 dataset under 416 × 416 pixels’ resolution.

Methods	Activation	FDL	Precision (%)	Recall (%)	F_1_ (%)	AP (%)
YOLOv3	Leaky	× 3	96.3	95.9	96.1	97.1
YOLOv4	Mish + SPP	× 3	98.0	100.0	99.0	99.0
YOLO-Tomato-A	Leaky	× 3	96.1	98.5	97.3	98.3
YOLO-Tomato-B	Mish	× 1	95.6	98.9	97.2	99.2
YOLO-Tomato-C	Mish + SPP	× 2	96.4	99.5	97.9	99.4

From the results in Tables 1, 2 and 3, under the presupposition, we found that the performance of all methods is very high due to the use of small datasets. This requires future investigation. Meanwhile, it is no doubt that the applied LWYS technique contributed to the excellent performance of the models.

There are variations in the evaluated performance between the methods. The compared results of AP within the tables show that YOLO-Tomato-A increased by 0.4% in Table 1, 1.2% in Table 2, and 1.2% in Table 3 from YOLOv3 model. This is due to features enhancement provided by DenseNet that make the model better at detecting small tomatoes. The activation of Mish in the models showed an increase in Precision, Recall, F_1_-score and AP. Taking it all from YOLOv3, the AP of YOLO-Tomato-B increased by 1.5% and YOLO-Tomato-C increased by 1.7% in Table 1, YOLO-Tomato-B increased by 2.1% and YOLO-Tomato-C increased by 2.3% in Table 2, and YOLO-Tomato-B increased by 2.2% and YOLO-Tomato-C increased by 2.4% in Table 3. YOLOv4 and YOLO-Tomato-C model in Tables 1 and 2 showed little or significant difference. However, Table 3 showed that the AP of YOLO-Tomato-C was slightly increased by 0.4% with 1.1% decrease in F_1_ score compared to YOLOv4. AP is more accurate than the F_1_ scores, because it considers the Precision-Recall relation globally. This is an indication that YOLO-Tomato-C is more accurate than YOLOv4 model in Table 3. The obtained model performance of Table 1 with respect to AP is more than Tables 2 and 3 due to high image quality.

We noticed little or no significant difference between the detection time of Raw, 0.5 ratio and 0.25 ratio, because they possess the same configuration file. With this, the detection time of YOLO-Tomato model per image on average were calculated as displayed in Table 4. The test results show that it takes an average of 45.3 ms for YOLOv3 model to count the both ripe and unripe tomatoes per frame image compared to YOLO-Tomato-A with 48.1 ms. This is an indication of tradeoff between accuracy and speed, because the incorporation of DenseNet into the network constituted an increase in accuracy with a decrease in detection speed. The same tradeoff was also found with YOLO-Tomato-B at 44.4 ms, YOLO-Tomato-C at 52.4 compared to YOLOv4 at 43.6 ms. SPP inclusion to YOLO-Tomato-C contributed to an increase in detection time. Meanwhile, the drastic detection time reduction experienced with YOLO-Tomato-B compared to YOLOv3 is due to the reduced FDL.

**Table 4:** Detection time difference between YOLO-Tomato models.

Methods	Activation	FDL	Time (ms)
YOLOv3	Leaky	× 3	45.3
YOLOv4	Mish + SPP	× 3	43.6
YOLO-Tomato-A	Leaky	× 3	48.1
YOLO-Tomato-B	Mish	× 1	44.2
YOLO-Tomato-C	Mish + SPP	× 2	52.4

### YOLO-Tomato visualization

**Figure 8:**
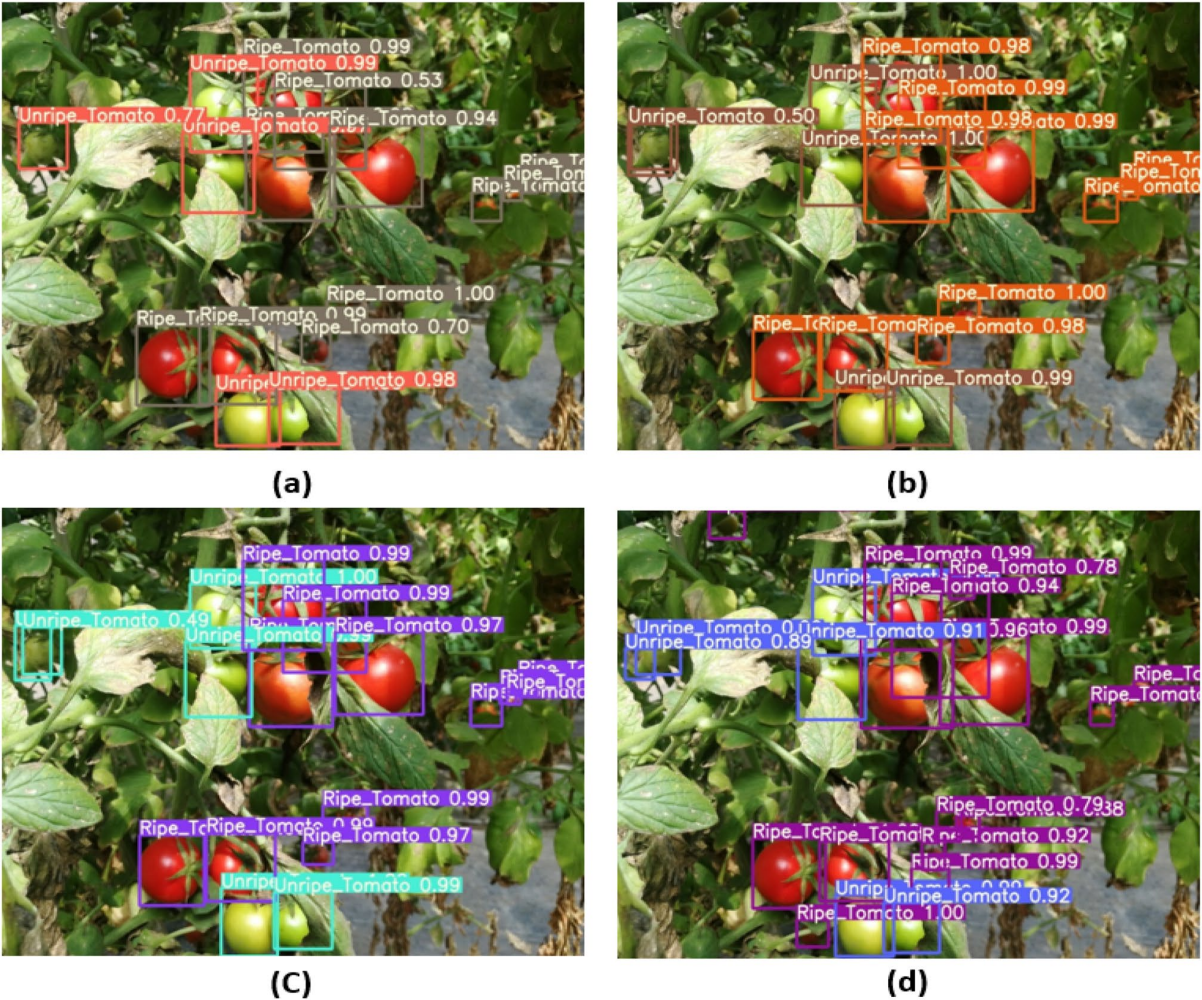
Comparison of YOLO-tomato models for (**a**) YOLOv3 (**b**) YOLO-tomato A (**c**) YOLO-tomato B, and (**d**) YOLO-tomato C detection results.

**Figure 9:**
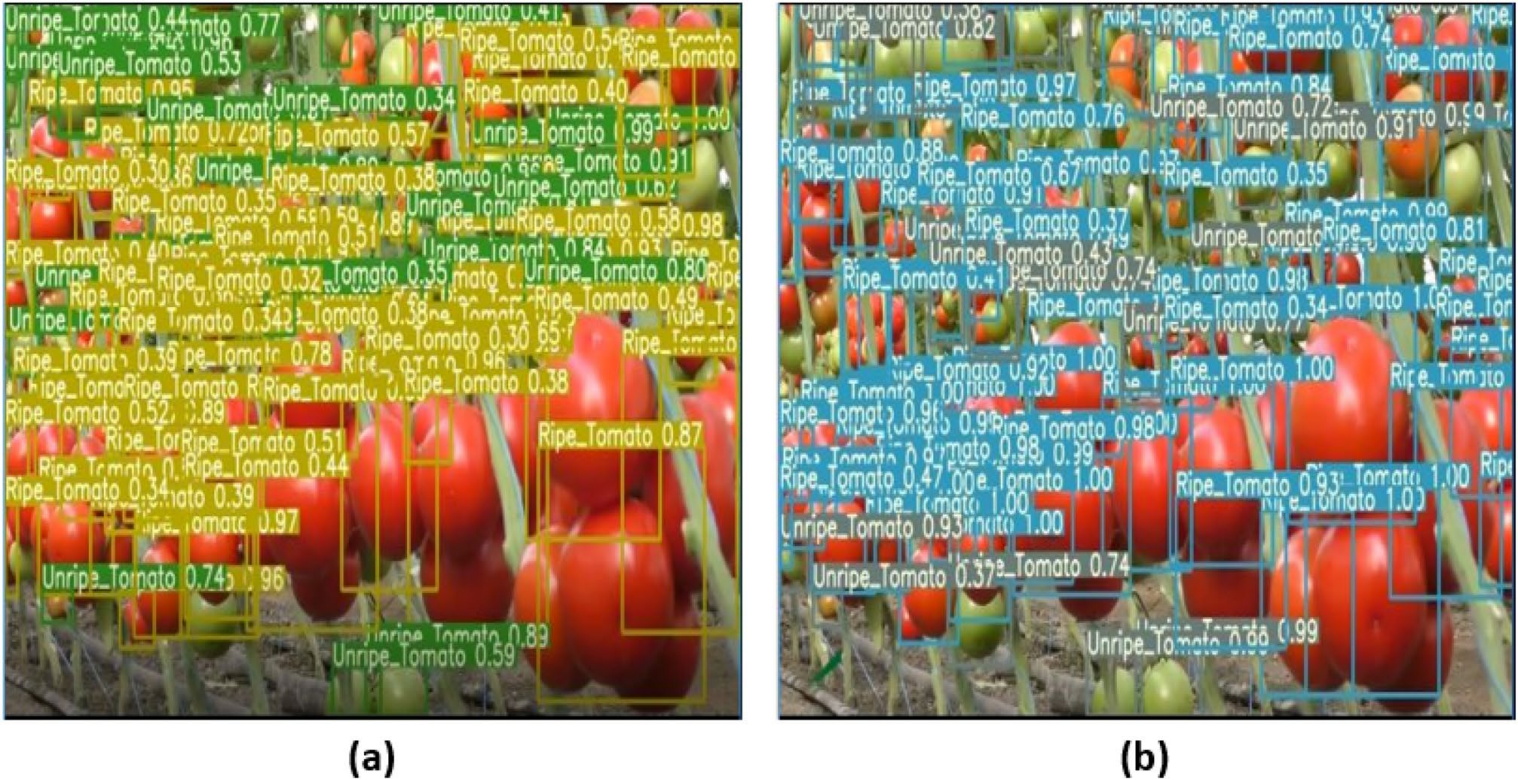
Comparison between the detection output of (**a**) YOLO-Tomato-C, and (**b**) YOLOv4.

The YOLO-Tomato visualization results in Figs. 8 and 9 were carried out to view the detected tomatoes with their percentages. The improvement in the model performance can be seen as the missed tomatoes detections in YOLOv3 (Fig. 8(a)) before modification is found in YOLO-Tomato-A (Fig. 8(b)) with an increased in percentage detection. Compared to YOLO-Tomato-A and YOLO-Tomato-B, the missed tomatoes detections in both were discovered by YOLO-Tomato-C in Fig. 8(d). This confirmed the importance of SPP to YOLO-Tomato-C in the reduction of missed detection and inaccuracies. Figure 9 showed little different between YOLOv4 and YOLO-Tomato-C, particularly with their percentage detections variation and spread of detections. In some cases, the percentage detections of YOLOv4 is a little higher than YOLO-Tomato-C, but the detected tomatoes spread of YOLO-Tomato-C is more than YOLOv4 model. This further proof the feature enhancement provided by SPP to YOLO-Tomato-C. It can be explained that the YOLO-Tomato-C model can be used to detect tomatoes with small and large targets.

### Different algorithms comparison

The average results of YOLO-Tomato model taken from Tables 1, 2 and 3 were compared with other state-of-the-art detection methods—YOLOv2^19^, YOLOv3^20^, Faster R-CNN^14^, and YOLO-Tomato^8^ for performance validation. The Precision, Recall, F_1_ score, AP, and Time of detections shown in Table 5 are applied for the comparison.

**Table 5:** Comparison of different tomato detection methods.

Methods	Precision (%)	Recall (%)	F_1_ (%)	AP (%)	Time (ms)
YOLOv2^19^	87.2	86.2	86.7	88.5	30
YOLOv3^20^	91.6	90.9	91.2	94.1	45
Faster R-CNN^14^	92.9	91.8	92.4	94.4	231
YOLO-Tomato^8^	94.8	93.1	93.9	96.4	54
YOLO-Tomato-A	95.9	99.1	97.5	98.3	48
YOLO-Tomato-B	95.4	99.1	97.2	99.3	44
YOLO-Tomato-C	96.4	99.6	97.9	99.5	52

The YOLO-Tomato models—YOLO-Tomato-A with AP 98.3%, YOLO-Tomato-B with AP 99.2%, and YOLO-Tomato-C with AP 99.4% shows the best detection performance among all the methods. These methods achieved the highest Recall, Precision, and F_1_ score compared to YOLOv2^19^, YOLOv3^20^, YOLO-Tomato^8^ and Faster R-CNN^14^, indicating the superiority of the proposed methods. The detection time of YOLO-Tomato-C is 52 ms per image on average, which is about 179 ms less than Faster R-CNN and the lowest among the three YOLO-Tomato models. This is an indication that our YOLO-Tomato models could perform tomato detection in real time with better generalization, which is important for harvesting robots.

## Conclusions

This research work proposed the use of YOLO-Tomato models for tomato detection, based on modified YOLOv3 model. The use of small tomato datasets obtained from complex environment condition to limit deep learning drawbacks, label what you see (LWYS) approach, densely architecture incorporated into YOLOv3 to facilitate reuse of features for well generalize tomato detection, Mish activation and spatial pyramid pooling (SPP) to reduce missed detections and inaccuracies are all adopted to make the detector as intelligent as humans. The experimental results show that the proposed methods performed better than other state-of-the-art methods with reference to average precision (AP) in particular. The level of YOLO-Tomato models’ performance increases as YOLO-Tomato-C > YOLO-Tomato-B > YOLO-Tomato-A with reference to average precision (AP), while the detection speed of YOLO-Tomato-B > YOLO-Tomato-A > YOLO-Tomato-C. In all, the YOLO-Tomato models show better generalization and real-time tomatoes’ detection, which is applicable for harvesting robots.
